# Making gametes from alternate sources of stem cells: past, present and future

**DOI:** 10.1186/s12958-017-0308-8

**Published:** 2017-11-16

**Authors:** Deepa Bhartiya, Sandhya Anand, Hiren Patel, Seema Parte

**Affiliations:** Stem Cell Biology Department, ICMR-National Institute for Research in Reproductive Health, Jehangir Merwanji Street, Parel, Mumbai, 400 012 India

**Keywords:** Embryonic stem cells, Induced pluripotent stem cells, Very small embryonic-like stem cells, Mesenchymal stromal cells, Niche, Gametes, Ovary, Testis

## Abstract

Infertile couples including cancer survivors stand to benefit from gametes differentiated from embryonic or induced pluripotent stem (ES/iPS) cells. It remains challenging to convert human ES/iPS cells into primordial germ-like cells (PGCLCs) *en route* to obtaining gametes. Considerable success was achieved in 2016 to obtain fertile offspring starting with mouse ES/iPS cells, however the specification of human ES/iPS cells into PGCLCs in vitro is still not achieved. Human ES cells will not yield patient-specific gametes unless and until hES cells are derived by somatic cell nuclear transfer (therapeutic cloning) whereas iPS cells retain the residual epigenetic memory of the somatic cells from which they are derived and also harbor genomic and mitochondrial DNA mutations. Thus, they may not be ideal starting material to produce autologus gametes, especially for aged couples. Pluripotent, very small embryonic-like stem cells (VSELs) have been reported in adult tissues including gonads, are relatively quiescent in nature, survive oncotherapy and can be detected in aged, non-functional gonads. Being developmentally equivalent to PGCs (natural precursors to gametes), VSELs spontaneously differentiate into gametes in vitro. It is also being understood that gonadal stem cells niche is compromised by oncotherapy and with age. Improving the gonadal somatic niche could regenerate non-functional gonads from endogenous VSELs to restore fertility. Niche cells (Sertoli/mesenchymal cells) can be directly transplanted and restore gonadal function by providing paracrine support to endogenous VSELs. This strategy has been successful in several mice studies already and resulted in live birth in a woman with pre-mature ovarian failure.

## Background

### Alternate sources of stem cells to make gametes in vitro

Making gametes in a Petri dish by directed differentiation of human pluripotent embryonic and induced pluripotent stem cells (hES/iPS) is considered one of the most important goals of stem cells research to help infertile couples attain biological parenthood. Research efforts by several groups across the globe have been focused to use ES cells grown in a Petri dish to differentiate into gametes for almost 3–4 decades based on when mouse [1, 2] and human [3] ES cells were first reported; and almost a decade of iPS cells research as they were first reported in 2006 [4]. Since primordial germ cells (PGCs) that arise from the epiblast- stage embryo are the natural precursors to the gametes [5, 6], the first crucial step involves conversion of ES/iPS cells into functional PGC-like cells (PGCLCs) which then spontaneously differentiate into gametes in vitro or when appropriate niche is provided in vivo. This conversion of pluripotent stem cells into PGCLCs remains a big challenge and Hayashi’s group successfully converted mouse ES/iPS cells into PGCLCs [7] whereas specification of human ES/iPS cells into PGCLCs still remains challenging [8, 9]. Efforts are also ongoing to convert primed human ES cells into naïve state to enhance their differentiation ability, as the naïve human ES/iPS cells may be better starting material to make human PGCLCs [10, 11]. This is because whereas mES/iPS cells exist in naïve state, hES/iPS cells are primed in nature being closer to stem cells derived from mouse epiblast state embryo, which do not exhibit any potential to differentiate into the germ cell fate. Research efforts are also directed to convert adult stem cells including spermatogonial stem cells (SSCs) and ovarian stem cells (OSCs) into gametes. Readers may refer to few recent reviews in the field [12–15].

There exists an additional novel population of pluripotent stem cells termed very small embryonic-like stem cells (VSELs) in all adult organs including testis and ovary, which can also be differentiated into gametes in vitro. Pluripotent VSELs in reproductive tissues were recently reviewed [16] and reasons why they have remained elusive so far were discussed [17]. It is well understood that during early development, PGCs migrate to the gonadal ridge where they differentiate into germ cells and cease to exist thereafter. However, it has been suggested that PGCs migrate not only to the gonads but to all developing organs and survive in few numbers throughout life [18]. There exists a developmental link between PGCs and VSELs in adult tissues and this explains why VSELs in hematopoietic system also express pituitary and sex hormone receptors [19]. Shaikh et al. [20] reported that mouse bone marrow VSELs besides exhibiting the ability to differentiate into 3 germ layers in vitro, in agreement with other reports [18, 21, 22], also differentiate into hematopoietic stem cells and germ cells when conducive culture conditions are provided. When cultured on a Sertoli cell bed and in the presence of Sertoli cells conditioned medium and FSH, VSELs differentiated into germ cells that expressed DAZL, STRA8 and GFRA and transcripts of Gfra, Vasa and Dazl [20]. Similar ability of SSEA1+ pluripotent stem cells from bone marrow to differentiate into male germ cells was recently reported by another group [23]. VSELs have been reported in adult testis [24–27], ovary [28–32] and uterus [33, 34]; express receptors for follicle stimulating hormone and survive oncotherapy due to their quiescent nature.

Being developmentally equivalent to PGCs, which are natural precursors to the gametes, VSELs are attractive alternative, pluripotent stem cells in adult gonads to obtain gametes. However, VSELs are not yet widely accepted by the reproductive biologists because of their small size and scarce nature. A recent update on fertility preservation published in September 2017 [35] does not even acknowledge VSELs and this is the main reason for compiling the present review article and compare them with other existing options on which research is ongoing to help infertile couples. Work needs to progress in different directions to ultimately help infertile couples including cancer survivors to achieve biological parenthood. We have earlier discussed that VSELs may be ideal stem cells to differentiate into gametes [36, 37] and that use of VSELs to attain biological parenthood may help avoid legal, ethical and safety issues associated with oncofertility [38]. Year 2016 was remarkable in that significant strides were taken in the field to differentiate stem cells into gametes. An update on various alternative sources of stem cells and progress made using them to obtain sperm and oocytes in vitro is provided in the review.

## Making sperm in a dish

### Differentiation of hES/iPS cells into male germ cells/sperm in vitro

Geijsen et al. [39] isolated SSEA1+/OCT4+ PGCs from mouse ES cells derived embryoid bodies. Treating these PGCs with retinoic acid resulted in the derivation of continuously growing embryonic germ cell lines that also showed erasure of methylation marks. These cells underwent further differentiation into functional haploid male gametes which when injected into oocytes resulted in the development of blastocysts. Nayernia’s group reported differentiation of mouse ES cells into sperm resulting in the birth of fertile offspring [40]. The mES cells differentiated into SSCs, which underwent meiosis in vitro, generated haploid sperm which were capable of fertilizing mouse oocytes after ICSI and resulted in the birth of live pups. However, a follow up publication showing hES cells also exhibit similar ability to produce sperm in vitro [41] was later retracted. Later research efforts were focused to first convert mES cells into PGCLCs and Hayashi et al. [7] transplanted mES cells derived PGCLCs into testicular environment that led to the formation of haploid sperm. But none of these studies demonstrated functionality of the in vitro derived gametes. Equizabel et al. [42] reported complete differentiation of human iPS cells into post-meiotic germ cells. iPS cells derived from various sources (skin, cord blood) on a human foreskin fibroblast feeder support successfully differentiated into germ cells. They discussed that iPS cells are epigenetically better predisposed to differentiate into germ cells as they may harbor some predisposing epigenetic memory of the original somatic cell in agreement with earlier reports [43]. Zhou et al. [44] first successfully reproduced complete maturation of mES cells derived PGCLCs that were further cultured on a testicular somatic cells support with sequential exposure to morphogens and sex hormones could reproduce meiosis resulting in haploid spermatid, which also resulted in viable and fertile offspring after ICSI. Highlight of the study was that they could track various events during differentiation in a very nice manner. Meiosis was tracked in vitro by studying sequential expression of specific markers. Initially chromosomal synapsis and DNA double stranded breaks and their resolution by homologous recombination repair monitored by studying expression of SPO11 and RAD51. Expression pattern of phosphorylated H2A histone family member X recapitulated meiosis progression as it was broadly distributed throughout the nucleus on D8 reflecting an association with double stranded breaks in DNA and later focal appearance on sex chromosomes suggested completion of synapsis. The nucleus showed expression of SYCP1 and SYCP3. Later an up-regulation of meiotic markers Dmc1, Stra8 and Sycp3 was observed by D10 followed by up-regulation of transcripts specific for haploid cells including Prm1, haprin and acrosin. However, success rate to obtain pups using gametes derived from mES cells of 1.89–6.67% is much lower compared to 9.46% in controls. Medrano et al. [45] converted human fibroblasts directly into haploid cells by over expression of 6 germ-line related factors. Whole genome analysis of spermatogonial cells obtained by differentiation of mES cells revealed aberrant methylation pattern, which could restrain their spermatogenic potential [46]. Saitou and Miyauchi [15] suggested that the epigenetic status of the differentiated gametes and embryos obtained by Zhou et al. [44] and Medrano et al. [45] need to be further investigated. The inefficient outcome and emerging data highlight safety issues associated with in vitro differentiated gametes from ES/iPS cells.

### Differentiation of VSELs into male germ cells/sperm in vitro

Our group has reported VSELs in adult human [24] and mouse [25–27] testis. Other groups have suggested possible presence of pluripotent stem cells as a sub-population among SSCs in adult human testis as well. Lim et al. [47] reported that SSEA-4 (pluripotent marker) and GFRA (marker for spermatogonial stem cells) do not co-express in testicular cells; Izadyar et al. [48] reported a sub-population among SSCs with pluripotent characteristics; Stimpfel et al. [49] reported pluripotent markers in frozen testicular biopsies of azoospermic men and Virant-Klun et al. [50] reported SSEA-4 positive stem cells in testicular tissue biopsies of azoospermic men by FACS analysis. For almost a decade between 2004 and 2014, lot of excitement was generated when different groups described ES-like colonies on culture of testicular tissue and it was proposed that testicular stem cells are the only stem cells in the body that can spontaneously reprogram to pluripotent state (discussed in details in [16]). Rather than reprogramming of SSCs, it is the VSELs that possibly grow as ES-like colonies as discussed earlier [51].These VSELs were found to survive oncotherapy in mouse [25–27] and human [52] testis. Kurkure et al. [52] have reported presence of VSELs along with Sertoli cells in the azoospermic testicular tissue biopsies of survivors of different kinds of childhood cancers subjected to various regimens of oncotherapy. The underlying reason for quiescence of VSELs lies in their epigenetic status and developmental origin. Being an overlapping population of PGCs, VSELs exhibit biallelic expression of imprinted genes including high levels of H19 and no IGF2 [53]. Every time a VSELs undergoes asymmetric cell division to self-renew and give rise to a SSC, genomic imprinting with increased (monoallelic) expression of IGF2 and reduced levels of H19 occurs in the SSC. As a result SSCs have greater ability to divide and undergo clonal expansion and do not survive chemotherapy. Patel and Bhartiya [27] proposed that VSELs are the most primitive stem cells in the testes that undergo asymmetric cell division to self-renew and give rise to the SSCs that further differentiate into germ cells, undergo meiosis to produce sperm. Anand et al. [25, 26] showed that VSELs and GFRA positive stem cells exist as two distinct populations of stem cells in the testis. VSELs are smaller in size, with a surface phenotype of LIN-CD45-SCA-1+ in mouse testis with nuclear OCT-4 whereas SSCs express cytoplasmic OCT-4. VSELs can be isolated as SSEA-1 positive stem cells from mouse testis and as SSEA-4 positive stem cells from human testicular tissue. Thus, the endogenous VSELs that survive oncotherapy could be targeted and strategies developed to differentiate them into sperm.

VSELs that survive chemotherapy were reported to spontaneously differentiate into sperm [54] since they are developmentally equivalent to the PGCs – natural precursors to gametes as discussed above. No growth factors were added exogenously to support differentiation/meiosis. Culture of seminiferous tubular cells from busulphan (25 mg/Kg) treated mouse was carried out using Sertoli cells conditioned medium and in the presence of FSH. Sertoli cells attached to the bottom of the culture dish to form a feeder layer whereas small sized, spherical VSELs, that survived chemotherapy were present singly at the start of culture. Over next few days, stem cells were observed to undergo asymmetric, symmetric cell divisions and clonal expansion. Three weeks culture resulted in the spontaneous differentiation of sperm and various stages of spermiogenesis were also documented. However, as the initial cells were not purified, it was possible that few mature stem/germ cells including SSCs existed in the initial cultures that could have differentiated into sperm. The approaches of in vitro differentiation of mouse VSELs by our group [54] and mES cells by Zhou et al. [44] were compared and discussed [55].

To address the above mentioned limitation of possible contamination with mature germ cells, Shaikh et al. [22] documented that VSELs purified from mouse bone marrow also differentiate into male germ cells that expressed DAZL, STRA8 and GFRA when cultured on a Sertoli cells bed and in the presence of Sertoli cells conditioned medium. The differentiated germ cells also expressed transcripts of Gfra, Vasa and Dazl. There was no chance of contaminating germ cells being present in the bone marrow. In the same month, April 2017 an article published by Nayernia’s group [23] also reported that SSEA-1positive, pluripotent VSELs from mouse bone marrow have the ability to differentiate into male germ cells that express markers specific for primordial germ cells, spermatogonial stem cells and spermatogonia including Mvh, fragilis, Dppa3, Stra8, DAZL, Piwil2, b1, and a6-integrins as well as meiotic-specific marker SYCP3. They had treated the cells with retinoic acid and cultured for longer time whereas our group [22] only added FSH and used Sertoli cells conditioned medium.

### Other approaches to obtain sperm in vitro

Galdon et al. [56] published an exhaustive systemic review describing the research efforts over last 50 years to achieve spermatogenesis in vitro. Reuter et al. [57] also reviewed progress on spermatogenesis using male germ cells in vitro using testicular tissue fragments, single cell suspensions or three-dimensional culture environments. Sato et al. in 2011 published two articles wherein they successfully cultured gonocytes/primitive spermatogonia in neonatal mouse testis over 2 months positioned at gas-liquid interphase [58]. Spermatids/ sperm obtained in vitro resulted in fertile offspring. They also developed a technology termed in vitro transplantation whereby they transplanted germ stem cells into removed tubules of genetically infertile mice (W/W^w^) in vitro [59]. The transplanted cells colonized, proliferated and differentiated into spermatid and sperm. The detailed protocols were later published describing differentiation of germline stem cells into sperm in an organ culture – a process that took about 6 weeks in vitro [60]. Elhija et al. [61] could produce sperm in vitro starting with immature mouse testis whereas Hileihel et al. [62] used juvenile Rhesus monkey testicular cells and obtained spermatids in vitro. However, lot more needs to be done to bring this to the clinics.

#### On the crossroads to treat male infertility

Semen preservation prior to oncotherapy in male cancer patients is advocated for fertility preservation and testicular tissue biopsies are cryopreserved for young pre-pubertal boys [63]. The field of using cryopreserved testicular tissue for fertility restoration has not developed as impressively as restoring fertility in females from cryopreserved ovarian tissue. Briefly, the cryopreserved testicular tissue could either be a source of germ cells (i) for intra-testicular transplantation (ii) for in vitro maturation of sperm (iii) autologus grafting for obtaining sperm for assisted reproduction. No clinical outcomes have been reported using cryopreserved testicular tissue and it may be another decade for successful fertility restoration using banked testicular tissue [35, 64].

It is evident from above review that it will be a long time before we could obtain human sperm starting with ES/iPS cells whereas human VSELs need to be differentiated in vitro into sperm. Another major concern is the highly inefficient nature of the process and pregnancy outcome in mice using gametes differentiated in vitro. Our group has further proposed that rather than isolating VSELs from azoospermic testicular biopsies and differentiating into sperm in vitro, a better approach will be to manipulate the VSELs that survive oncotherapy to restore spermatogenesis in vivo [65]. Anand et al. [26] showed that besides resulting in germ cells loss and infertility, busulphan treatment affects the Sertoli cells transcriptome also. On transplanting GFP positive Sertoli cells/ mesenchymal cells that provide the niche to testicular stem cells, it was observed that the transplanted cells assembled as neo-tubules and possibly provided a paracrine support to the surviving VSELs in adjacent, native azoospermic tubules to undergo spermatogenesis and regenerate the chemoablated testis. A similar approach of transplanting mesenchymal cells in chemoablated testis has been reported by several groups [66–74] resulting in restoration of testicular function and live births (Table 1) although none of these studies acknowledge the presence of VSELs.

**Table 1:** List of studies reporting beneficial effects of transplanting mesenchymal cells via different routes to restore spermatogenesis after chemoablation

Study	Highlights
Vahdati et al. [66]	The study evaluated the regenerative effect of bone marrow derived stem cells (CD29+/CD73+/CD45-) on spermatogenesis of busulphan treated infertile hamster. Following 60 days after efferent duct injection of bone marrow stem cells, histological evaluation of testis showed presence ofspermatogonia, primary spermatocytes, spermatids and sperm in seminiferous tubules compared to negative controls.
Maghen et al. [67]	The study evaluated the role of human umbilical cord blood MSCs in regeneration of testicular niche. In addition to supporting the expression of murine germ cells and putative SSCs in vitro, the in vivo potential was evaluated by transplantation of MSCs into azoospermic mouse model. Transplanted cells majorly localized into interstitial space. Following transplantation, partial reconstruction of seminiferous tubule architecture was observed.
Abd Allah et al. [68]	The study evaluated the effect of transplantation of human cord blood derived mesenchymal cells (CD34-) and hematopoietic stem cells (CD34+) by local injection into testis of busulphan treated recipient mice. Testicular histoarchitecture was found normal and sperm were present in lumen in mesenchymal cells transplanted group compared to the HSC transplanted group. l
Ghasemzadeh-Hasankolaei et al. [69]	The study evaluated the fate of autologous bone marrow MSCs (isolated and labelled with PKH26) after transplantation into testes of busulphan treated Wistar rats. Transplanted bone marrow MSCs were studied at 3 time points (4,6 and 8 weeks) after transplantationand were found to survive post transplantation as studied by PKH26 expression. Some transplanted cells homed at germinal epithelium and expressed germ cell markers DAZLand STELLAindicating differentiation to spermatogonia.
Rahmanifar et al. [70]	The study aimed to evaluate the seminiferous tubules of azoospermic rats following bone marrow derived MSCs transplantation. The recipient mice were prepared by busulphan treatment. Following efferent duct injection of MSCs, the transplanted tubules showed spermatogenesis with presence of germinal cells like spermatogonia, primary spermatocytes, spermatids and sperm.
Anand et al. [25, 26]	The study showed that VSELs survive busulphan treatment in the testis and resume spermatogenesis when mesenchymal/Sertoli cells are transplanted through the intertubular route.
Chen et al. [71]	In vivo differentiation potential of human cord blood mesenchymal stem cells was evaluated following transplantation into busulphan treated mice seminiferous tubules. Transplanted tubules exhibited improved histology compared to busulphan treated tubules.
Yang et al. [72	The study investigated the potential of human umbilical cord MSCs (CD31−/CD73+/CD105+) to promote spermatogenesis regeneration in busulphan treated testis following interstitial injections. Three weeks after injection, there was an increased expression of meiotic markers namely, Dazl, Vasa, Stra8, Scp3, Cyclin A1, Tnp2, Pgk2, Miwi, Tex18 and Akap3. Protein level expression of MIWI, VASAa and SCPwas also increased compared to controls.
Sabbaghi et al. [73]	Rat bone marrow MSCs (5-10 × 10^6^ cells) were cultured and transplanted via rete testis into torsioned azoospermic testis. Germ cell specific markers (OCT4, VASAand c-KIT) were monitored for the differentiation of MSCs after transplantation.
Aziz et al. [74]	Bone marrow derived MSCs were transplanted into busulphan treated rats. Results showed that MSCs have potential for in vivo transdifferentiation into spermatids and spermatocytes.

These results of restoring spermatogenesis by transplanting niche cells are ready to initiate clinical trials and will bring about a paradigm shift in our current approach to oncofertility. There may be no need to cryopreserve testicular tissue from pre-pubertal individuals prior to oncotherapy nor to be concerned about infertility as a side effect. Providing healthy niche cells to non-functional testis could restore testicular function. But well-planned clinical studies need to be undertaken to confirm the beneficial effects observed in mice for possible translation to humans. This will involve transplanting autologus mesenchymal cells (from any source including bone marrow) into the azoospermic testis via inter-tubular route to study the effect. Few trials are registered using this approach (https://clinicaltrials.gov/ct2/show/ NCT02041910) but the outcome is still awaited.

## Making oocytes in a dish

### Differentiation of hES/iPS cells into female germ cells/oocytes in vitro

Hans Scholer’s group was the first to suggest that pluripotent stem cells could differentiate into gametes [75]. Although the functionality was not proved, the authors showed formation of oocytes and follicle-like structures in vitro starting with both female and male mES cells. Embryonic stem cells were coaxed to undergo spontaneous differentiation to form embryoid bodies from which PGCs were isolated, expanded in vitro and differentiated. Later Hayashi et al. [76] used female mES/iPS cells and induced them into PGCLCs, which were then aggregated with ovarian somatic cells and on transplantation under the ovarian bursa, gave rise to GV stage oocytes that successfully contributed to the formation of fertile offspring after IVM and IVF. However, the process to obtain pups from in vitro PGCLCs derived oocytes was less efficient (approximately 3.9%) compared to in vivo PGC derived (approximately 12.7%) oocytes. Almost half of the zygotes that were obtained from the PGCLCs derived oocytes had 3PN with 2 maternal and 1 paternal chromosomes. They failed to extrude polar body. Later Hayashi and Saitou [77] published their protocols and discussed the existing limitations that need to be overcome for the robust generation of mature gametes or for application of the culture system to other species, including humans and livestock [9, 78].

The differentiation of PGCLCs and PGCs to obtain oocytes in vitro was reviewed [14, 79] and highlighted low competence of in vitro produced oocytes starting with PGCLCs. Two major success stories were reported in 2016. Complete in vitro generation of fertile oocytes starting with PGCs isolated from female fetal gonads collected on 12.5 dpc was reported [80]. Various stages during oogenesis like meiosis, oocyte growth and genomic imprinting were reproduced in vitro. It led to the formation of fertile offspring – up to7 from a cultured gonad. The frequency of development of normal fertile pups from 2-cell stage embryos starting with PGCs to derive oocytes was comparable to that observed after natural delivery in mice. Hikabe et al. [81] could reconstitute whole process of oogenesis in vitro from mES and iPS cells (derived from fetal and adult tail tip fibroblasts) to generate eggs which were further used to derive pluripotent stem cell lines. However, they reported 3.5% success rate using mES/iPS cells derived gametes compared to 61.7% using in vivo generated gametes. These results clearly suggest that the in vivo developed PGCs and stem cell derived PGCLCs in vitro are not identical and was discussed by Ge et al. [82]. Converting ES/iPS cells into PGCLCs involves extensive epigenetic changes [83] which may be difficult to attain in vitro. Hayashi et al. [84] published their protocols to produce functional primordial germ cells in vitro, which further differentiate into oocytes and healthy pups.

### Differentiation of VSELs into female germ cells/oocyte-like structures in vitro

VSELs are located in the ovary surface epithelium along with OSCs [85].Virant-Klun’s group has initially reported in vitro differentiation of human ovarian small sized (2–4 um), pluripotent stem cells, obtained by gentle scraping of ovary surface epithelium (OSE), into oocyte-like structures [28, 29]. Later, Parte et al. [30] detected similar small sized VSELs along with slightly bigger OSCs among the ovary surface epithelial cells smears which on 3 weeks culture, spontaneously differentiated into oocyte-like structures. The ovary surface epithelial cells attach at the bottom of the culture dish to form a feeder layer whereas VSELs are observed to undergo proliferation/ differentiation into oocyte-like structures. Later, Parte et al. [86] observed that various processes like formation of germ cell nests, Balbiani body-like structures and cytoplasmic streaming extensively described during fetal ovary development, were indeed well recapitulated during in vitro oogenesis in adult OSE cultures along with characteristic expression of stem/germ cell/oocyte markers. Sriraman et al. [31] reported similar stem cells in mouse OSE cells collected by enzymatic digestion. These stem cells survived chemotherapy and could differentiate in vitro into oocyte-like structures. VSELs were recently reported by another group in adult mouse ovaries and their differentiation into oocyte-like structures in vitro [32]. However, none of these studies have tested the functional competence of these oocyte-like structures that differentiate from the VSELs.

Similar to mouse testis, VSELs survive chemotherapy in mouse ovaries also [31]. VSELs were quantitated by flow cytometry as small sized cells (3–5 μm) with a surface phenotype of LIN−/CD45−/SCA-1+ in normal adult (0.02 ± 0.008%) and chemoablated (0.03 ± 0.017%) ovaries and further treating chemoablated mice with FSH resulted in further increase of VSELs (0.08 ± 0.03%). Table 2 is a compilation of various studies reporting beneficial effects of transplanting mesenchymal cells in chemoablated ovaries. A human baby has also been born by transplanting autologus, bone marrow derived mesenchymal cells directing into the non-functional ovaries of a patient with POF [87].

**Table 2:** Various studies reporting beneficial effects of mesenchymal cells to restore ovarian function after chemoablation in animal models

Study	Highlights
Wang et al. [107]	Menstrual blood mono-nuclear cells were transplanted via intra-peritoneal route into mice chemoablated with cisplatin. The transplanted cells localized into ovarian interstitium. Following transplantation, follicle numbers increased and levels of sex hormones reached normalcy and improved ovarian function.
Fouad et al. [108]	The study compared the efficiency of human amniotic membrane and adipose derived MSCs following transplantation into cyclophosphamide induced ovarian failure. Transplantation of MSCs from both sources showed increased number of follicles and oocytes alongwith increase in serum estradiol and decrease in serum FSH compared to chemoablated controls. The efficacy was more using human amniotic membrane MSCs.
Song et al. [109]	In cyclophosphamide induced POF rat model human umbilical cord MSCs were transplanted by using either tail intravenous injection or injection into ovary in situ. Following transplantation, folliculogenesis was recovered along with hormonal secretions and decreased ovarian cell apoptosis.
Kilic et al. [110]	Transplantation of bone marrow MSCs into cyclophosphamide treated rat showed protective effects by reducing germ cell apoptosis and DNA damage. Increased primordial follicular counts were obtained following transplantation compared to controls.
Liu et al. [111]	Bone marrow derived MSCs were transplanted into POF rat model obtained by cisplatin treatment. The transplanted group showed increase in follicle growth and estradiol levels compared to control, thereby restoring ovarian structure and function.
Liu et al. [112]	Transplantation of human endometrial MSCs into POF mouse model induced by cyclophosphamide showed survival of transplanted cells in ovaries and upregulation of ovarian markers along with increased estradiol and follicle number compared to control and restoring ovarian function.
Lai et al. [113]	Following transplantation of skin derived MSCs from male/female mice into recipient mice with busulphan and cyclophosphamide induced ovarian damage, partial restoration of fertility was observed. Transplanted MSCs grafted into host ovaries and increased expression of oogenesis markers was observed compared to controls.
Abd Allah et al. [114]	MSCs from bone marrow of male rabbits were injected intravenously into female rabbits chemoablated with cyclophosphamide. Increase in follicle numbers and resumption of normal follicular structure was observed compared to controls by histology. Ovarian tissues showed presence of Y-chromosome containing donor cells indicating engrafting of transplanted cells.
Wang et al. [115]	Umbilical cord MSCs were transplanted intravenously into POF mouse model made by cyclophosphamide treatment. Reduced apoptosis in cumulus cells, increased number of follicles and recovery of ovarian function was observed.
Liu et al. [116]	Transplantation of human amniotic fluid cells (CD44+/CD105+) showed survival into cyclophosphamide induced POF mice models for atleast 3 weeks following transplantation and proliferated.
Selesniemi et al. [117]	Bone marrow mono-nuclear cells from young adult female mice (with EGFP transgene under β-actin) were transplanted into young/middle aged females. Following this treatment, the fertile potential of the aging female was sustained for longer period than the normal reproductive senescence. Offsprings did not show EGFP expression. However, donor bone marrow derived somatic cells accumulate in recipients indicating efficient donor cell engraftment.
Fu et al. [118]	Effect of MSC transplantation on ovarian damage induced by chemotherapy using cyclophosphamide in rats was studied. Cultured MSCs were labelled with GFP and transplanted directly into bilateral ovaries. Following transplantation, the ovarian function was improved. Reduced germ cell apoptosis and upregulation of Bcl-2 was found in vivo.

### Other approaches to obtain oocyte-like structures in vitro

Besides VSELs, OSCs also exist in the adult ovary (similar to VSELs and SSCs in the testis) and have been extensively used to generate primordial follicles using innovative strategies. In fact OSCs were detected and published initially in a landmark paper by Tilly’s group in 2004 [88]. Pluripotent SSEA4 positive VSELs were reported later in adult ovary [28, 29]. Indeed two populations of stem cells exist in ovary surface epithelium and VSELs with nuclear OCT-4 differentiate and give rise to OSCs with cytoplasmic OCT-4 [85, 89].

Zou et al. [90] established a neonatal mouse FGSC line, cultured for more than 15 months, FGSCs from adult mouse were maintained for more than 6 months. These stem cells were GFP labeled and on transplantation in ovaries of infertile mice underwent oogenesis and GFP positive offspring. Later, Tilly’s group successfully expanded DDX-4 positive stem cells in vitro for months from both mouse and human ovarian cortex [91]. Injecting GFP positive human OSCs into human ovarian cortical biopsies leads to assembly of primordial follicles when transplanted in immuno-deficient female mice –resulted in complete assembly of primordial follicles. These protocols were later published [92].

‘Eggbert’ was born by culturing primordial follicles isolated from newborn mice in vitro using a two-step culture method. The pup however, developed health problems including obesity and neurological abnormalities [93]. Later the protocol was further modified and another 59 pups were born to provide company to ‘Eggbert’ [94]. But the process remained inefficient. Of 1160 2-cells embryos transferred, only 66 (5.7%) developed to term and 7 pups (10.6%) died at birth. The remaining 59 pups (27 females, 32 males) survived to adulthood. By comparison, of 437 transferred two-cell stage embryos derived from in vivo-grown oocytes, 76 (17.4%) developed to term and 4 (5.3%) died at birth. The remaining 72 pups (35 females, 37 males) survived to adulthood. Shen et al. [95] transplanted fetal 12.5 dpc ovaries under the kidney capsule that initiated oocyte growth from the pre-meiotic germ cells. Subsequently, the primary and early secondary follicles generated in the ovarian grafts were isolated and cultured for 16 days in vitro. The mature oocytes ovulated from these follicles were able to fertilize in vitro to produce live offspring. Offspring born by IVF were normal and able to successfully mate with both females and males. The patterns of the methylated sites of the in vitro mature oocytes were similar to those of normal mice.

Another alternative is to develop artificial ovary that implies 3D culture of preantral, immature follicles in a scaffold which could be a source of oocytes upon transplantation [96]. This approach could negate introduction of malignant cells. Woodruff’s group developed a two-step follicle culture strategy that recapitulated the dynamic human follicle growth environment in vitro [97]. Follicles developed from the preantral to antral stage and produced meiotically competent metaphase II oocytes after in vitro maturation. Later Kniazeva et al. [98] reported live births on transplanting primordial to primary follicles from young ovaries encapsulated in fibrin beads. The group recently developed a bioprosthetic ovary using microporus scaffolds to restore ovarian function in bilaterally ovariectomized mice. Follicles matured, ovulated, secreted hormones, formed corpus luteum that secreted progesterone to support pregnancy and resulted in birth of fertile pups [99].

#### On the crossroads to treat female infertility

It is evident from the above description that research is progressing on several fronts to produce gametes and provide fertility options to women with premature ovarian failure including cancer survivors. Human ES/iPS cells will require more research to reach the clinics whereas a baby has already been born by transplanting autologus mesenchymal cells in the non-functional ovary [87] presumably by new follicle developed from endogenous VSELs that exist in POF ovaries.

Besides banking oocytes and embryos in adult females prior to oncotherapy, ovarian cortical tissue slices are cryopreserved in young and unmarried girls and also if the cancer is hormone sensitive and oncologists cannot wait to initiate treatment [35, 100]. Generally, one whole ovary gets removed and cortex is cryopreserved as thin slices. The cortical tissue slices are later transplanted back on the surface of the non-functional ovary as a source of follicles for fertility restoration. Besides the theoretical risk of reintroducing malignant cells, the transplanted cortical tissue slices have limited lifespan and the process is highly invasive in nature. As of today almost 86 babies have been born and 9 ongoing pregnancies [101]. But the question remains as to *whether the transplanted cortical tissue slices are a source of oocytes or do they induce regeneration of the intact non-functional ovary or both*.

We believe that the transplanted cryopreserved ovarian tissue could also regenerate the non-functional ovary. There are several lines of evidence to support this. Oktay et al. [102] reported that subcutaneous transplantation of ovarian cortical tissue slices at a heterotypic site in a female survivor of Hodgkin lymphoma resulted in four spontaneous pregnancies and three live births. The woman was earlier rendered menopausal due to preconditioning chemotherapy before bone marrow transplantation, had elevated levels of FSH 46.4 to 96.6 mIU/ml and hot flashes. The group discussed that possibly the microenvironment of the non-functional ovary gets destroyed by chemotherapy and paracrine/endocrine signals provided by the transplanted cortical tissue result in regeneration of the ovary from the bone marrow or resident stem cells. Based on recent advances on ovarian stem cells research, it is being understood that the niche gets disrupted by chemotherapy and also with age [103–106]. Resident stem cells can regenerate the non-functional ovary when healthy niche (source of growth factors and cytokines) is provided. Aged, non-functional mouse ovaries were made functional on transplanting in a young host [103] stressing on the fact that niche gets compromised with age and that a healthy, young niche is crucial for stem cells function and oocyte development. Sriraman et al. [31] have shown that VSELs survive chemotherapy in mouse ovary and later increase in numbers in response to FSH treatment. Similarly Virant-Klun et al. [28, 29, 50] have reported presence of pluripotent, very small sized SSEA4 positive stem cells in postmenopausal and POF human ovaries. Edessey et al. [87] transplanted autologus mesenchymal cells in a POF ovary and a healthy baby has been born. Presumably the mesenchymal cells provided a healthy paracrine support to the resident stem cells resulting in oocyte development followed by the birth of a baby. Several groups have conducted similar studies [107–118] in mice wherein transplantation of mesenchymal cells has restored ovarian function and also live births (Table 2). VSELs that survive oncotherapy in the non-functional ovary possibly undergo neo-oogenesis and follicle assembly when a healthy paracrine support is provided by transplanted cortical tissue pieces as suggested earlier [107] or after transplanting mesenchymal cells (Table 2). Current understanding on a role of VSELs/OSCs resulting in neo-oogenesis and follicular assembly was recently reviewed [119, 120]. We have reported similar findings in the testis including that testicular niche (Sertoli cells) gets compromised after chemotherapy and transplanting mesenchymal cells restores spermatogenesis [26, 55].

These results in females (Table 2) are very similar to those shown in Table 1 and discussed above. It becomes crucial to take cognizance of the findings that a novel population of stem cells termed VSELs exist in adult gonads and survive oncotherapy whereas the stem cells niche gets compromised with age and also as a result of oncotherapy. Providing a healthy niche by transplanting mesenchymal cells can regenerate both non-functional testis [55] and ovary [121].

## Obtaining gametes from non-gonadal sources

It is intriguing to note that several somatic organs [22, 23, 40, 122–131] have been reported as a source of gametes (Table 3) including bone marrow, skin, amniotic fluid, menstrual blood and also pancreatic stem cell line. This does not come as a surprise to us since VSELs exist in all adult/fetal tissues and being pluripotent have the ability to differentiate into any kind of cell types based on the cues provided. Both our group [22] and Shirazi et al. [23] have reported ability of VSELs/SSEA-1 positive cells from the bone marrow to differentiate into male germ cells in vitro. Similarly, Tilly’s group reported female germ cell markers in mouse bone marrow [129].

**Table 3:** List of published reports describing extra-gonadal source of gametes

References	Extra-gonadal sources from which gametes have been obtained
Female	
Lai et al. [122]	Human Menstrual blood
Ge et al. [123]	Human skin cells
Yu et al. [124]	Human amniotic fluid
Dyce et al. [125]	Porcine fetal skin
Lee et al. [126]	Mouse BM and PB
Dyce et al. [127]	Clonal pancreatic stem cell line
Dyce et al. [128]	Fetal and new born porcine/mouse/human skin
Johnson et al. [129]	Mouse bone marrow and peripheral blood
Male	
Shirazi et al. [23]	Adult mouse bone marrow
Shaikh et al. [22]	Adult mouse bone marrow
Hua et al. [130]	Human fetal bone marrow
Drusenheimer et al. [131]	Human bone marrow
Nayernia et al. [40]	Mouse bone marrow

## Discussion and conclusions

**Fig. 1:**
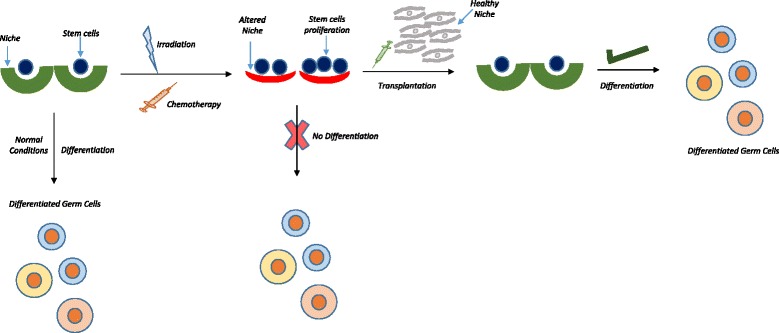
Novel strategy to restore gonadal function. Stem cells exist in various tissues including testis and ovary in close association with their niche which controls their fate. Niche factors support gene expression, proliferation and differentiation of stem cells thereby maintaining tissue homeostasis. When the gonads (ovary or testis) are exposed to cytotoxic injury, radio- or chemo-therapy, ‘true’ stem cells survive the insult (due to their quiescent nature) and rather increase in numbers [25–27, 31] in an attempt to restore homeostasis. However, the niche undergoes irreversible changes due to the insult. As a result, although stem cells exist; they are unable to differentiate and restore gonadal function. On transplanting niche cells (mesenchymal stromal cells or Sertoli cells), surviving VSELs differentiate and thus restore gonadal function [25, 26]. This strategy of manipulating endogenous stem cells to regenerate non-functional gonads after oncotherapy is far superior to making gametes in vitro. Transplanting mesenchymal stromal cells (Tables 1 and 2) results in positive outcome in animal studies and was confirmed by a recent meta-analysis [141]. A baby girl has been born by transplanting mesenchymal cells in POF ovary [91]. This successful strategy obviates the need to differentiate gametes in vitro and cryo-banking of gonadal tissue prior to oncotherapy. [VSELs: Very small embryonic-like stem cells]

Research needs to progress in various directions to make gametes and help infertile couples including cancer survivors to attain biological parenthood. Changing life style has resulted in delayed childbearing and also individuals who undergo gender reassignment require fertility options. Considerable progress has been made and still lot more time is required to obtain gametes from ES/iPS cells for clinical use. Cryopreserved ovarian cortical tissue transplantation has been largely successful however use of cryopreserved pre-pubertal testicular tissue has not yet reached the clinics. At this juncture, present review offers a novel alternative to restore gonadal function from endogenous stem cells by providing them a healthy niche. This is achieved by transplanting autologus mesenchymal cells in the non-functional gonads and gametes will be developed in vivo from endogenous VSELs. Pros and cons of various approaches to address infertility are mentioned in (Fig. 1) Table 4 [132–138]. While this review was being compiled, Fazeli et al. [139] published a systemic meta-analysis describing the beneficial effects of transplanting mesenchymal cells to restore fertility in chemoablated gonads. It is crucial for the scientific community to first arrive at a consensus on existence of VSELs in adult gonads and then appreciate their potential to restore gametogenesis on transplanting mesenchymal cells. They will be a game changer in the field of oncofertility.

**Table 4:** Critical evaluation of various options available to tackle infertility

Various approaches	Current status
Making Artificial Gametes from ES/iPS Cells	• Protocols are not yet available to convert human ES/iPS cells into PGCLCs. Obtaining gametes from mouse ES/iPS cells is successful but still inefficient and has associated epigenetic concerns. • Human ES cells exist in primed state and have to be converted to naïve state for further differentiation • Gametes obtained from human ES cells will not ensure biological parenthood unless and until the hES cells are derived by somatic cell nuclear transfer (therapeutic cloning). However, despite recent success in the field [130], this may also not be a practical solution since the hES cells derived by this method will have to be first converted into PGCLCs which is a road-block at present. • Serious concern exists with the use of iPS cells to make gametes. They exhibit genomic [131, 132] and mitochondrial [133] DNA mutations which seriously limit their clinical utility [134]. They also retain residual epigenetic memory of the somatic cell from which they are derived [135, 136]. Even for other clinical conditions, it is being advocated to use allogeneic over autologus iPS cells as it is more practical and safe. The first clinical trial for macular degeneration using autologus iPS cells was suspended due to safety concerns [137]. But use of allogeneic iPS cells to make gametes will not ensure biological parenthood. • Gametes obtained from hES/iPS cells will require use of assisted reproductive technology to have a baby. But this approach is expensive and inefficient.
Restoring Fertility by Targeting Endogenous, Resident Stem Cells (VSELs)	• Major advantage of VSELs compared to hES/iPS cells is that they are equivalent to PGCs. Developmentally they are obtained from epiblast-stage embryo and thus relatively more mature compared to ES cells obtained from the inner cell mass of blastocyst stage embryo. • Being equivalent to PGCs (natural precursors to gametes), VSELs spontaneously differentiate into sperm & oocytes in vitro • VSELs survive oncotherapy in the gonads and transplanting niche cells (autologus mesenchymal cells from any source) can regenerate non-functional, azoospermic testis and POF ovary. This approach will ensure restoration of normal fertility. If mesenchymal cells are transplanted in pediatric cancer survivors, the gonads could serve as a source of hormones for secondary sexual development and later on in life will also be a source of gametes. • This approach could circumvent all associated safety and epigenetic concerns that invariably creep in when cells are cultured in vitro. Normal fertility will be restored and there will be no need of use of assisted reproductive technology. This approach is more feasible and less expensive. Most importantly, this approach could obviate the need to cryopreserve gonadal tissue prior to oncotherapy.
Other Available Options	• All the below mentioned alternatives are still being researched upon and are not yet ready for the clinics • In vitro follicle culture • Artificial ovary • Entire ovary cryopreservation • Use of feto-protective agents • In vitro culture of OSCs and SSCs • Transplantation of cryopreserved cortical tissue pieces has given promising results. However, it is an invasive and expensive procedure. • Only option available is semen banking for men. Use of cryopreserved testicular tissue is not yet available in the clinics

VSELs were reported for the first time in 2006 by Ratajczak’s group and are pluripotent stem cells in adult tissues. They have been extensively studied in hematopoietic system and survive total body irradiation in mice [140] similar to their survival in chemoablated as well as aged gonads. Our work highlights that it is the stem cells niche which gets compromised and needs to be improved to achieve regeneration of non-functional gonads after oncotherapy and also could improve aged gonads. Being pluripotent, they possess the ability to differentiate into 3 germ layers in vitro and have the true potential to regenerate diseased adult tissues including gonads. Rather than ceasing to exist in the developing gonads, primordial germ cells survive in various adult organs including the gonads in few numbers. This has been suggested by others as well [141] and also an overlap of hematopoietic and germ cells has been reported [142, 143]. VSELs can best be studied in chemoablated testis and ovary which are devoid of all other germ cells and are located in the basal layer of seminiferous epithelium in the testis and in the ovary surface epithelium. Being very small in size, they have been discarded over time while processing cells for various experiments [17]. Their developmental origin and equivalence to PGCs explains their ability to spontaneously differentiate into gametes in vitro. To conclude, we are reminded of a sentence from Lord of Flies by William Golding 'The greatest ideas are the simplest'.
